# NFκB1 inhibits memory formation and supports effector function of ILC2s in memory-driven asthma

**DOI:** 10.3389/fimmu.2023.1217776

**Published:** 2023-07-27

**Authors:** Mukesh Verma, Divya Verma, Anand Santosh Sripada, Kapil Sirohi, Rangati Varma, Anita Sahu, Rafeul Alam

**Affiliations:** ^1^ Division of Allergy & Immunology, Department of Medicine, National Jewish Health, Denver, CO, United States; ^2^ School of Medicine, University of Colorado Denver, Denver, CO, United States

**Keywords:** asthma, IL2s, memory, IL33, NFκB1, RUNX1

## Abstract

**Background:**

ILC2s are capable of generating memory. The mechanism of memory induction and memory-driven effector function (trained immunity) in ILC2s is unknown.

**Objective:**

NFκB1 is preferentially expressed at a high level in ILC2s. We examined the role of NFkB1 in memory induction and memory-driven effector function in a mouse model of asthma.

**Methods:**

Intranasal administration of Alternaria, flexivent, ELISA, histology, real-time PCR, western blot, flow cytometry and immunofluorescence staining.

**Results:**

NFκB1 was essential for the effector phase of memory-driven asthma. NFκB1 was critical for IL33 production, ILC2 generation, and production of type-2 cytokines, which resulted in eosinophilic inflammation and other features of asthma. NFκB1 induction of type-2 cytokines in ILC2s was independent of GATA3. NFκB1 was important for allergen induction of ILC3s and FoxP3+ Tregs. NFκB1 did not affect Th2 cells or their cytokine production. In contrast to its protagonistic role in the effector phase, NFκB1 had an antagonistic role in the memory phase. NFκB1 inhibited allergen-induced upregulation of memory-associated repressor and preparedness genes in ILC2s. NFκB1 upregulated RUNX1. NFκB1 formed a heterodimer with RUNX1 in ILC2s.

**Conclusions:**

NFκB1 positively regulated the effector phase but inhibited the induction phase of memory. The foregoing pointed to an interdependent antagonism between the memory induction and the memory effector processes. The NFκB1-RUNX1 heterodimer represented a non-canonical transcriptional activator of type-2 cytokines in ILC2s.

## Introduction

Innate immune cells mount an immediate response to an environmental insult in order to protect the host. This response is aimed at destroying and eliminating the insult. Repetitive insults trigger the formation of memory, and generate trained immunity (1–5). This memory/trained immunity can be recalled by a repeat exposure. The recall response is usually stronger and occurs with a subthreshold dose. We previously reported the development of a mouse model of ILC2 memory and memory-driven asthma (1). The latter is a manifestation of the effector phase of memory. The mechanistic processes involved in memory formation and execution of the effector phase is poorly understood. The induction of memory in ILC2s is associated with increased expression of a number of genes that we categorize into two programs—a gene repression program (Nr4a2, Bach2, Zeb1, and JunD) and a preparedness program (Fhl2, FosB, Stat6, Srebf2 and Mpp7). The gene repression program comprises genes that are well-known repressors and inducers of memory in T cells and NK cells (6–10). The repressors mark and repress previously activated genes, which constitutes the molecular/epigenetic mechanism of memory. All four repression program genes regulate cytokine production. Nr4a2 (Nurr1) additionally induces FoxP3 (10). The Bach2 DNA binding motif overlaps with that of the AP1 motif. Consequently, Bach2 antagonizes gene activation by AP1 (8). JunD is a member of the AP1 transcription factor and inhibits AP1 function by heterodimerizing with a transcriptionally active Fos subfamily member (7). Zeb1 is important for survival of memory CD4 T cells (6). The repressors are regulated by the preparedness-associated molecules. Fhl2 negatively regulates JunD and Bach2. FosB is a transcription factor of the AP1 family and heterodimerizes typically with Jun family members. Stat6 is a transcription factor for type-2 cytokines. Srebf2 is a master transcription factor for lipid synthesis genes that are important for metabolic fitness. The preparedness program is primed but not activated. Its activation upon a recall allergen challenge downregulates the repressors—Bach2 and JunD, and activates the ERK1/2-AP1 and the STAT6 pathways to elicit the memory-driven asthma phenotype.

We observed in the memory model an increase in Nfκb1, which was of interest to us for the following reasons. ILC2s expressed relatively high levels of Nfκb1 after allergen sensitization and a recall challenge when compared to lung macrophages, dendritic and NK cells (1). This is in agreement with the Immgen database (www.immgen.org). The Nfκb1 gene encodes the protein p105, which undergoes proteasomal processing to generates p50 (11). The protein p50 usually binds to p65 (RelA) to form the canonical NFκB heterodimer that drives the expression of pro-inflammatory genes. Both p65 and p50 have DNA binding domains; but, only p65 has a transactivation domain. p50 can homodimerize and bind to the NFκB recognition site but is unable to initiate gene transcription, and acts as a repressor (11). Hence, NFκB1 could function in the gene repression program in memory ILC2s by forming a homodimer and functioning as a repressor. It could also function as a transcriptional activator by forming a heterodimer with a transactivation domain-containing partner either in the preparedness program of the memory phase or in the effector phase. Because of this potential dichotomous function, we examined the role of NFκB1 in the memory phase and the effector phase of trained immunity of ILC2s in a mouse model of asthma.

Our study showed that NFκB1 supported the effector phase of trained immunity but inhibited the gene repression program and the preparedness program in the memory induction phase. Germline deletion of Nfκb1 resulted in loss of the effector functions–airway hyperreactivity (AHR), type 2 inflammation and IL33 expression, but increased expression of the memory phase-associated repressor and preparedness genes.

## Methods

### Mouse studies

The animal protocol for this study was approved by the National Jewish Health IACUC. We used B6.Cg-Nfkb1tm1Bal/J from the Jackson Laboratory (JAX stock #006097). This strain is commonly known as p50- or Nfκb1 knockout (Nfκb1-/-). We used littermate, and in select experiments, C57BL/6 as wild-type controls.

### Mucosal sensitization of mice

**Figure 1:**
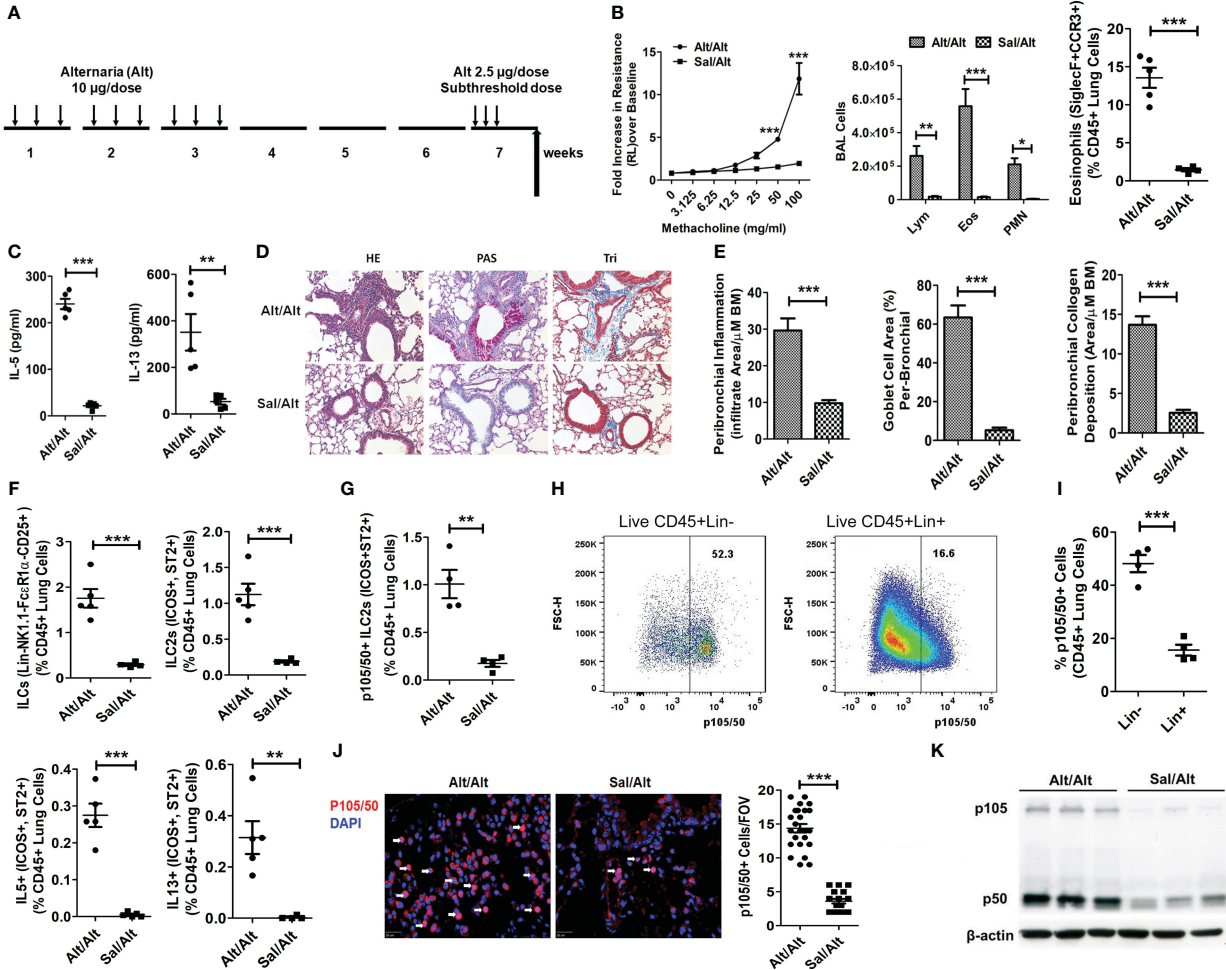
ILC2s and NFκB1 in a mouse model of memory-driven asthma. **(A)** A schematic diagram of the timeline of allergen exposure, recall challenge and experiments. Groups of C57BL/6 j female mice were intranasally exposed to extract of *Alternaria* (Alt) (10 ug in 20 uL of saline/dose) or saline alone (Sal) 3 alternate days per week in week 1-3 and then rested for 3 weeks. Both groups had a recall challenge in week 7 with a subthreshold dose (2.5 ug/dose) of Alt on three consecutive days and then examined for airway hyperreactivity (AHR), inflammatory and immunologic alterations 3 days later. **(B)** Increase in lung resistance over the baseline (as measured by Flexivent) in response to increasing doses of inhaled methacholine in Alt/Alt and Sal/Alt groups, differential leukocyte counts of bronchoalveolar lavage (BAL), and the frequency of eosinophils (CD45+Siglec8+CCR3+ cells) in the lung from the study groups. *** P<0.0001, **P<0.001 and *P<0.05 vs control group, 2way ANOVA or unpaired t test, N=4-5/group. **(C)** IL5 and IL13 (measured by ELISA) in BAL. ***P<0.0001 and **P<0.001, unpaired t test, N=4-5/group. **(D, E)** H&E staining for airway inflammation, PAS staining for goblet cells and trichrome staining for peribronchial collagen deposition, and their morphometric quantification from the study groups (Alt/Alt and Sal/Alt), unpaired t test, N=4/5 per group; infiltrate area/µM BM: inflammatory cell area per micrometer of the basement membrane. **(F)** Total live lung ILCs (CD45+NK1.1-FcϵR1α- Lin-CD25+), ILC2s (CD45+NK1.1-FcϵR1α- Lin-CD25+ ICOS+ ST2+), IL5+ and IL13+ ILC2s (CD45+NK1.1-FcϵR1α- Lin-CD25+ ICOS+ ST2+) in the study groups by FCM (Flow cytometry). ***P<0.0001 and **P<0.001, unpaired t test, N=4-5/group. **(G)** p105/50+ILC2s (CD45+NK1.1-FcϵR1α- Lin-CD25+ ICOS+ ST2+ cells). ***P<0.0001, N=4-5/group. **(H, I)** Representative p105/50 flow plots for Lin- and Lin+ cells, and their quantification. **(J)** Immunofluorescence staining for p105/50 nuclear localization and quantification ***P<0.0001 (FOV: Field of View), unpaired t test, N=4-5/group. **(K)** Western blot for p105/50 expression in the lung tissue from Alt/Alt and Sal/Alt mice (3 mice per group). These data are representative of 3 independent experiments with 4-5 mice per group. The white arrows point to the nuclear localization of p105/50.

We used the *Alternaria alternata* (Alt) allergen extract. Mice were sensitized to Alt as shown in **Figure 1**. Briefly, Alt (10 μg/dose), was administered in 20 ul volume intranasally on alternate days 3x a week for 3 consecutive weeks unless otherwise stated. The mice were then rested for 3 weeks. In week 7 they were given a recall challenge with a subthreshold dose of the sensitizing allergen on 3 consecutive days. The subthreshold recall dose was 2.5 ug/mouse. The mice were sacrificed 3 days later.

### Airway hyperreactivity measurement

The measurement of airway hyperreactivity in response to methacholine by Flexivent was described in details previously (13). Briefly, mice were anesthetized with ketamine (180 mg/kg), xylazine (9 mg/kg), and acepromazine (4 mg/kg). After loss of foot-pad pinch reflex, a tracheotomy was performed and the mouse was attached via an 18-gauge cannula to a small-animal ventilator with a computer-controlled piston (Flexivent; Scireq) (flexiVent Fx; SCIREQ, Montreal, Quebec, Canada). After performing initial calibrations (cylinder pressure channel and nebulizer calibration), we conducted dynamic tube calibration and used a default program called QuickPrime 3 (version 7) for measurement of airway resistance in response to methacholine. This program uses prime perturbations, which are a family of complex forced oscillation perturbations at a frequency greater than and less than the subject’s ventilation frequency (1-20.5 Hz). The amplitude of the oscillatory signal is preset to a volume that is slightly smaller than the subject’s tidal volume (0.2 mL). Volume and pressure signal are recorded during a measurement, and the flow signal is derived from the volume. The foregoing allows calculation of Newtonian resistance, tissue damping, tissue elastance, and hysteresivity. Resistance measurements were taken to establish the baseline for total lung resistance and at each methacholine dose. Group averages were expressed as the fold increase over baseline resistance (mean ± SEM) (13).

### Histology and imaging

Paraffin embedded lungs were sectioned and stained with hematoxylin and eosin (H&E) for morphometric analysis, PAS staining. For mucus and Mason’s trichrome for collagen deposition. We measured the entire peribronchial inflammatory area (infiltrate area). We also measured the perimeter of the basement membrane (BM) of the corresponding bronchus. We presented the data as the infiltrate area/μM BM. Images were acquired on a Nikon Eclipse TE2000-U microscope using 20x dry lenses at room temperature through a Diagnostics Instruments camera model #4.2 using the Spot software 5.0. H&E, PAS and trichrome sections were mounted using Permount medium. Images were adjusted for brightness and contrast to improve viewing (13)

### Thin-section immunofluorescence microscopy

Paraffin-embedded lung sections (4-µm thickness) were used for this study. Images were taken under polarized light using an upright dry 40× objective. Tissues were deparaffinized and after antigen retrieval, permeabilized with 0.4% triton X-100. Tissues were blocked with 10% BSA and maintained in PBS + 5% BSA + 0.4% triton X-100 throughout antibody treatments. Primary antibodies were incubated at 4°C overnight and secondary antibodies were incubated for 1 hr at room temperature. Primary antibodies used (1:200 dilution) include rabbit anti-NFκB p65 (#8242, Cells Signaling Technology); mouse anti-cRel (#MA5-15859, Thermo Fisher Scientific); rabbit anti-NFκB1 (#13586, Cells Signaling Technology); mouse anti-NFκB1 (#NBP2-66976, Novus); rabbit anti-RUNX1(#ab229482, Abcam); goat anti-IL33 (#AF3626 R&D); mouse anti-ICAM1 (#sc-1511, Santa Cruz Biotechnology, Inc); and mouse anti-CD3 ((# sc-7296, Santa Cruz Biotechnology, Inc). Goat anti-rabbit and anti-mouse IgG-A594 or IgG-A488 were used as secondary antibodies (1:200 dilution at RT). For anti-goat, rabbit anti-goat IgGA-647 was used as secondary antibody (1:200 dilution at RT). DAPI was used for nuclear counterstaining. The ProLong Gold antifade reagent was used as mounting media. Mount slides were examined with a Leica DM6000 B. Slide Book 6 (3i) was used for analysis and capturing images.

### Lung digestion for isolation of single cell preparations

Mouse lungs were perfused with saline and then subjected to mechanical mincing followed by digestion at 37°C for 45 minutes in RPMI with 10% FBS, 1% penicillin/streptomycin and collagenase Type I (1mg/mL) (Worthington # LS004197) as described previously (13). Isolated cell suspensions were agitated at room temperature for 10 minutes in RPMI with 100U/mL DNAse I prior to filtration through 40μm filters and red blood cell lysis. Single cell suspensions were either subsequently cultured in RPMI with 10% FBS, 1% penicillin/streptomycin at 37^0^C in CO2 incubator overnight or used immediately for staining for flow cytometry and analysis, depending on the experiment and schedules (13).

### Flow cytometric analyses of ILCs, eosinophils, neutrophils and other cells

The mouse lung single cell suspension was blocked with an anti-FcR blocking reagent for 10 min (minutes) at 4^0^C (Miltenyi Biotec; # 130-092-575) before staining with a fixable viability dye. Cells were then stained with surface receptor antibodies at 4^0^C for 30 min (cells were washed twice with staining buffer (PBS plus 1% BSA between each staining). Then we fixed the cells with 4% paraformaldehyde for 15 min at 4^0^C, washed them with the staining buffer (PBS plus 1% BSA), and incubated with the permeabilization buffer (PBS [pH 7.4] plus 1% BSA plus 0.1% saponin) at 4^0^C for 20 min. After centrifugation, we resuspended the cells in the permeabilization buffer and incubated with antibodies against the intracellular proteins (cytokines and transcription factors) at 4^0^C for 30 minutes. We washed the cells twice with the permeabilization buffer, resuspended in the staining buffer, and maintained at 4^0^C until flow analysis. For intercellular cytokines, we added monensin (2 mmol/L) to the cells and cultured for 4 hours before staining

Most of the fluorophore-conjugated antibodies used for flow cytometry were purchased from Biolegend, Inc. (San Diego, CA). Others were from eBioscience or R&D, Inc., unless otherwise stated. ILC cells were stain with FITC or BV605-labelled anti-CD45.2 (clone 104), pacific blue or Alexa Flour 700 -labeled lineage marker antibodies (CD3, Ly-6G/Ly-6C, CD11b, CD45R/B220, TER-119/Erythroid cells, pacific blue or Alexa Flour 700 FcϵR1α (Biolegend # 134314 or 134324), PerCP-Cy5.5-conjugated anti-CD25 (eBioscience; clone PC61.5), pacific blue or Alexa Flour anti-mouse NK-1.1 Antibody (Biolegend # 108722 or 156512)., APC or PE labelled anti-IL5 (TRFK5), and PE-Cy7 or PE labeled anti-IL13 (eBioscience; clone eBio13A). For eosinophil and neutrophil: Clone #245707), PerCP/Cy5.5 anti-mouse Ly-6G (Biolegend # 127616; clone1A8), PE/Cy7 anti-mouse CD11c (Biolegend # 117318; clone N418), Brilliant Violet 421™ anti-mouse/human CD11b (Biolegd# 101236; cloneM1/70), PE anti-mouse CD193(Biolegend # 144506; clone J073E5), Alexa Fluor^®^ 647 Rat Anti-Mouse Siglec-F (BD Pharmingen™# 562680; clone E50-2440). PerCP-eFluor^®^ 710 anti-GATA3 Antibody (eBioscience # 46-9966-42) and Brilliant Violet 605™ anti-mouse NK-1.1 Antibody, Fixable Viability Dye eFluor™ 780 (#65-0865-14 from eBioscience) and Zombie Aqua™ Fixable Viability Kit (#423102 from Biolegend) used for detection of cell viability. Stained cells were analyzed using the LSR Fortessa cell analyzer (BD). Flow data were analyzed with FlowJo version 10.0.7 software (Tree Star).

### Real time PCR

Total RNA was isolated from frozen lung samples using Trizol (Invitrogen). cDNA was synthesized using the Verso cDNA synthesis kit (Thermo Scientific # AB-1453/B) according to manufacturer’s instructions as described previously (13). Gene specific PCR products were amplified using the qPCR SYBR Green Rox mix (ThermoScientific # AB-4162/B) and primers outlined in online repository **Table S1**. Primers were designed using the Applied Biosystems 7000 Sequence Detection System software. The levels of target gene expression were normalized to 18S expression using the 2^-ΔCt^ method. The primer list is given in **Table S1**

### Western blotting and Immunoprecipitation

Lysates for extraction of protein and for immunoprecipitation (IP) were prepared using RIPA Lysis and extraction buffer # 89900 and Pierce IP Lysis buffer # 87787; Thermo Fisher Scientific, respectively, according to the manufacturer protocol. Protein concentration was determined by the BCA method (Pierce™ BCA Protein Assay Kit # 23225; Thermo Fisher Scientific). An aliquot of 20 µg the lysates was used for SDS– polyacrylamide gel electrophoresis (SDS-PAGE) and analyzed by immunoblotting. Blot was incubated overnight at 4°C with respective antibody; rabbit anti-NFκB p65 (#8242, Cells Signaling Technology); mouse anti-cRel (#MA5-15859, Thermo Fisher Scientific); rabbit anti-NFκB1 (#13586, Cells Signaling Technology); mouse anti-NFκB2 (# NBP2-66977, Novus); rabbit anti-RUNX1(#ab229482, Abacm); and detected using an HRP conjugated IgG antibody. For IP 100 µg of the lysates was incubated overnight at 4°C with an immunoprecipitating antibody (RUNX1 # LS-B13948; LSBio, NFκB1 (# 13586; Cell Signaling Technology) and β-actin (#12262; Cell Signaling Technology) along with an appropriate isotype control antibody. Protein A/G PLUS-Agarose (sc-2003; Santa Cruz Biotechnology, Inc) was added and kept at 4°C on a rotating platform for 2 h. Thereafter, the immune complexes were isolated, and separated by SDS—PAGE.

### ELISA for cytokines

IL5 (R&D#DY405-05) and IL13 (R&D#DY413-05) in the bronchoalveolar lavage fluid were measured by ELISA kits as per manufacturer’s instruction.

### Statistical analyses

Statistical analyses were performed, and data figures were prepared with GraphPad Prism software (version 6; GraphPad Software, San Diego, Calif). Statistical significance was analyzed by t test or ANOVA.

## Results

### Elevated level of type 2 cells and cytokines was due to increase of NFκB1

To understand the role of NFκB1 in allergen-induced memory ILC mediated asthma, we treated C57B/6 mice with *Alternaria* (Alt) and saline (Sal) as described previously (1). According to this protocol (**Figure 1A**), mice were intranasally exposed to 10 ug/dose of Alt or Sal 3 days a week for 3 consecutive weeks, rested for 3 weeks and then given a recall challenge with a subthreshold 2.5 ug/dose of Alt on 3 consecutive days. AHR and inflammatory indices were measured 3 days later. We found that Alt/Alt treated mice had increased AHR, and increased frequency of bronchoalveolar lavage (BAL) lymphocytes, eosinophils and neutrophils, and lung eosinophils as compared to Sal/Alt mice (**Figure 1B**). Cytokine measurement in BAL showed elevated levels of IL5 and IL13 (**Figure 1C**). Lung histology showed increased levels of peribronchial and perivascular inflammation, mucus-producing goblet cells, and airway remodeling in Alt/Alt mice (**Figures 1D, E**). Flow cytometric analysis of the lung cells showed an increased number as well as frequency of total ILCs (CD45+Lin-CD25+NK1.1- FcϵR1α-), ILC2s (CD45+Lin-CD25+NK1.1- FcϵR1α-ICOS+ ST2+) as well as type 2 cytokine (IL5 and IL13)+ ILC2s (**Figure 1F**; **Figure S1A**). The gating strategy for the flow analysis is shown in **Figures S1B–F**. NFκB1 was previously shown to be involved in regulation of ILC2, CD4+ ILC3, and NCR+ ILC3 (14). We checked its expression by flow cytometry in Alt/Alt and Sal/Sal treated mice. p105/50 was highly expressed in ILC2s and minimally in non-ILCs (Lin+ CD45+ cells) (**Figures 1G–I**). We checked p105/50 expression in NK, ILC1, ILC3, epithelial and CD4 cells. The frequency of p105/50+ cells was the highest in ILC2s as compared to other cells (**Figures S2A–D**). Next, we examined nuclear localization of p105/50 in the lung tissue by immunofluorescence staining. Alt/Alt mice showed a higher level of nuclear localization of p105/50 than Sal/Alt mice (**Figure 1J**). We confirmed p105/50 expression in lung cells from Alt/Alt mice by western blot (**Figure 1K**). This data indicated that increased AHR and type-2 inflammation in allergen treated mice were associated with increased expression of NFκB1.

### NFκB1 is important for the ILC2 mediated pathogenesis of allergen induced asthma.

**Figure 2:**
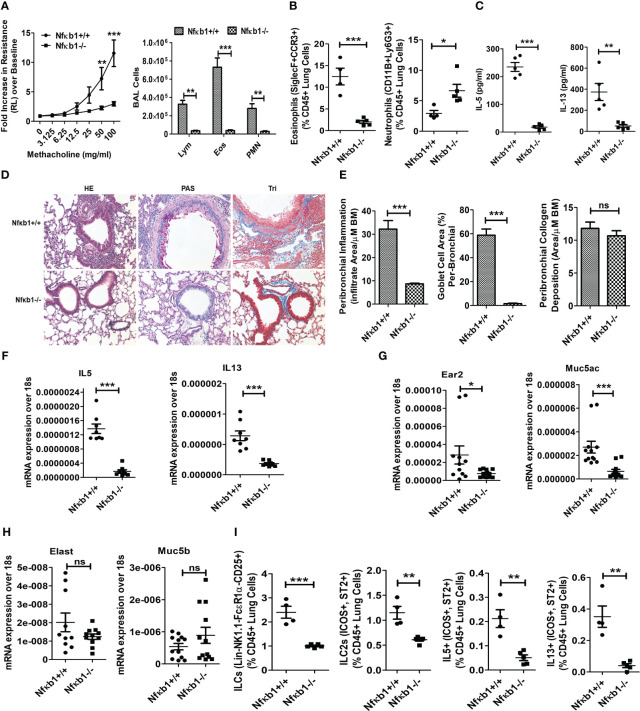
NFκB1 is important for memory ILC2-induced asthma. **(A)** Comparison of airway hyperreactivity and BAL lymphocytes, eosinophils and neutrophils between Nfκb1+/+ and Nfκb1-/- mice. ***P<0.0001 and **P<0.001, 2way ANOVA, N=4/5group, N=4/5group. **(B)** Lung eosinophils (CD45+SiglecF+ CCR3+ cells) and neutrophils (CD45+CD11B+ Ly6g+). ***P<0.0001, unpaired t test N=4-5/group. **(C)** IL5 and IL13 in BAL as measured by ELISA. ***P<0.0001 and **P<0.001, N=4-5/group. **(D, E)** H&E staining for airway inflammation, PAS staining for goblet cells, and trichrome staining for peribronchial collagen deposition, and their morphometric quantification. ***P<0.0001 and N=4-5/group. **(F-H)** qPCR analysis of mRNA for IL5 and IL13 **(F)**, Ear2 and Muc5ac **(G)**, Elastase (Elast) and Muc5b **(H)** from the lung tissue. *P<0.05, **P<0.001 and ***P<0.0001, N=8-10/group. **(I)** Total lung ILCs (CD45+NK1.1-FcϵR1α- Lin-CD25+), ILC2s (CD45+NK1.1-FcϵR1α- Lin-CD25+ ICOS+ ST2+), and IL5+ and IL13+ ILC2s (CD45+NK1.1-FcϵR1α- Lin-CD25+ ICOS+ ST2+) from the study groups. **P<0.001 and ***P<0.0001, N=4-5/group. All data are representative of 3 independent experiments. ns, not significant.

The role of NFκB1 in memory ILC2-mediated asthma is unknown. To address this question, we designed the following experiment. We sensitized Nfκb1+/+ (littermate) and Nfκb1-/- female mice intranasally with Alt according to **Figure 1A**. Nfκb1-/- mice sensitized to and recalled with Alt (Alt/Alt) had negligible AHR and reduced BAL lymphocytes, eosinophils and neutrophils as compared to Nfκb1+/+ mice (**Figure 2A**). Alt/Alt treated Nfκb1-/- mice had increased CD11b+Ly6G+ neutrophils and decreased Siglec F+ CCR3+ eosinophils in the lung as compared to Nfκb1+/+ mice (as measured by flow cytometry) (**Figure 2B**; **Figure S3A**). Nfκb1-/- mice had lower levels of IL5 and IL13 in BAL (ELISA) **(** **Figure 2C**). Lung histology showed reduced levels of peribronchial and perivascular inflammation, and mucus-producing goblet cells (**Figures 2D, E**). Airway remodeling was similar in Nfκb1-/- and Nfκb1+/+ mice (**Figures 2D, E**). We examined the expression of mRNA for select type-2 inflammation associated genes. The expression of mRNA for IL5 and IL13 was decreased in Nfκb1-/- mice (**Figure 2F**). The mRNA for eosinophil-associated ribonuclease A family member 2 (Ear2) and the mucin gene Muc5ac were lower while that for Muc5b and the neutrophil elastase were unchanged in knockout mice (**Figures 2G, H**). Flow cytometric analysis of the lung cells showed reduced frequency of total ILCs (CD45+Lin-CD25+NK1.1- FcϵR1α-), ILC2s (CD45+Lin-CD25+NK1.1- FcϵR1α-ICOS+ ST2+) and type 2 cytokine (IL5 and IL13)+ ILC2s (**Figure 2I**). The gating strategy for the flow analysis is shown in **Figure S3B**. We repeated this experiment using a group of male mice. We observed similar trends with Nfκb1-/- mice (**Figures S3C–G**). The results suggested that Nfκb1 was important for the effector phase of memory ILC2-driven asthma.

### NFκB1 affects Tregs, ILC3s and ILC2s but not CD4 T cells.

**Figure 3:**
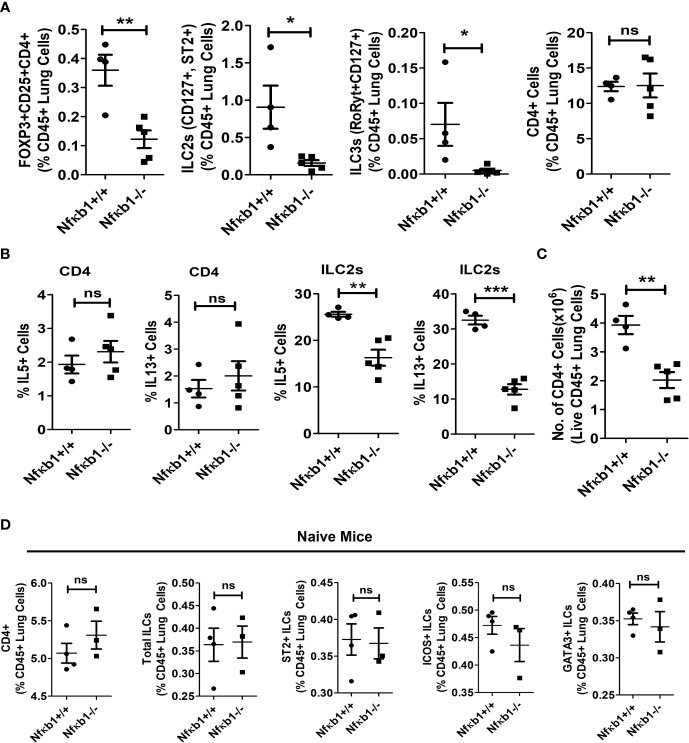
NFκB1 deletion affects Tregs, ILC3s and ILC2s but not CD4 T cells. **(A)** The frequency of lung CD4+Foxp3+CD25+ Tregs, CD127+ST2+ILC2s (CD45+NK1.1-FcϵR1α- Lin-CD25+), RoRyt+CD127+ILC3 (CD45+NK1.1-FcϵR1α- Lin-CD25+) and CD4 T cells presented as % CD45+ lung cells in Alt/Alt treated Nfκb1+/+ and Nfκb1-/- mice. **P<0.0001, *P<0.05, unpaired t test, N=4-5/group. **(B)** Frequency of IL5+ and IL13+ ILC2s and CD4 T cells in the lung from Nfκb1+/+ and Nfκb1-/- mice. **P<0.0001, ***P<0.0001, N=4-5/group. **(C)** The number of recovered total CD4 T cells from the lung obtained from the study groups. **P<0.0001, N=4-5/group. **(D)** The frequency of CD4+ T cells, total ILCs (lin-NK1.1-FcεRI-CD25+), and ST2+, ICOS+ and GATA3+ ILC2s (lin-NK1.1-FcεRI-CD25+) in naïve Nfκb1+/+ and Nfκb1-/- mice. N=3-4/group. These data are representative of 3 independent experiments. ns, not significant.

Next, we examined the effect of Nfκb1 deletion on Tregs, ILC3 and CD4 T cells. We previously reported that our chronic asthma model was associated with heightened numbers of CD4 T cells and CD4+ nTregs, and that CD4 T cells contributed to the increased magnitude of airway inflammation (13, 15). CD4+Foxp3+CD25+ Tregs, CD127+ST2+ILC2s and RoRyt+CD127+ILC3 but not CD4 T cells were reduced in Nfκb1-/- (**Figure 3A**). Likewise, IL5 and IL13+ ILC2s but not CD4 T cells were reduced in Nfκb1-/- mice (**Figure 3B**). Note that the frequency of IL5+ and IL13+ CD4 T cells was low in control wild type mice in our asthma model. We checked the total number of CD4+ T cells in the lung and found ~ 50% less CD4+ T cells in Nfκb1-/- mice (**Figure 3C**). Next, we asked whether the development of CD4 T cells and ILC2s was impaired in Nfκb1-/- mice. To address this question we studied naïve Nfκb1+/+ and Nfκb1-/- mice. The frequency of CD4+ T cells and CD25+, ST2+, ICOS+ and GATA3+ ILC2s was similar in both mouse strains. (**Figure 3D**; **Figure S4A**).

### NFκB1 regulation of type-2 inflammation-associated molecules

GATA3 is a signature transcription factor for type-2 T cells and ILCs. GATA3 expression and its mean florescence intensity (MFI) were similar in ILC2s and CD4 T cells from Nfκb1+/+ and Nfκb1-/- mice (**Figure 3D**; **Figure S4B**), which did not explain reduced cytokine production by ILC2s in Nfκb1-/- mice. Next, we studied RUNX1, another transcription factor that contributes to ILC2 cytokine production (16). Nfκb1-/- mice showed reduced expression of RUNX1 (**Figure S4C**).

TSLP, IL25 and IL33 are three major upstream cytokines for the type-2 immune response, and they are frequently elevated after an allergen exposure. We did not observe any significant difference in mRNA for IL25 and TSLP (**Figure S4D**). TSLPR+ ILC2s were reduced in Nfκb1-/- mice (**Figure S4E**). IL25R+ (IL17RE+) ILC2s were not detected (data not shown) by flow in our model. In contrast to IL25 and TSLP, IL33 was reduced at the mRNA and the protein level in Nfκb1-/- mice (**Figures S4F, G**). ICAM1 regulates inflammatory cell influx into the tissue. Since Nfκb1-/- mice had reduced inflammation, we studied ICAM1 expression. ICAM1 expression was reduced in Nfκb1-/- mice (**Figure S4H**). The foregoing findings suggested that Nfκb1 was important for induction of select transcription factors and adhesion molecules.

### The role of NFκB1 in ILC2 memory formation

**Figure 4:**
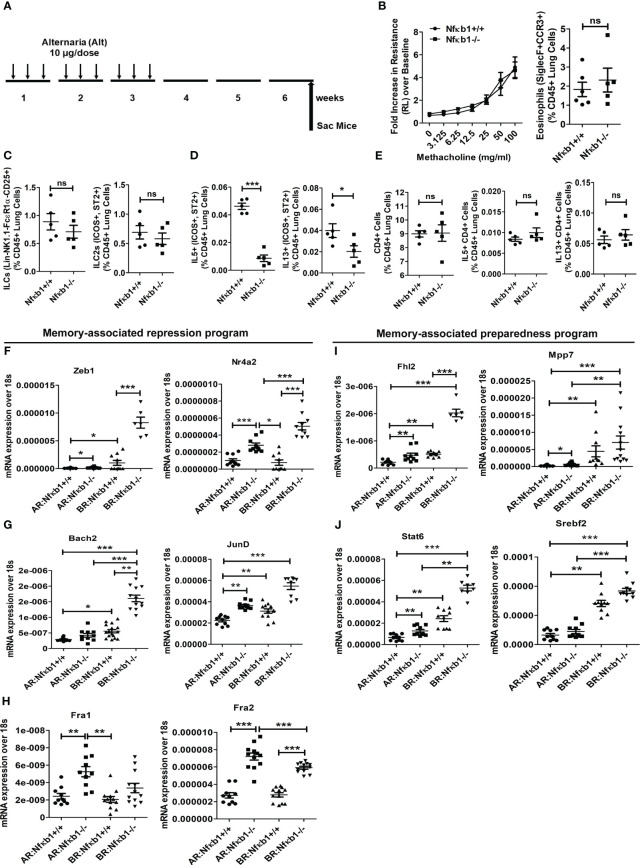
NFκB1 is important for the effector function but not for memory formation of ILC2s. **(A)** A time line of allergen exposure and experiments without a recall challenge (see **Figure 1** for reference) for panels B-E. **(B)** Comparison of airway hyperreactivity and lung eosinophils (CD45+SiglecF+ CCR3+) between Nfκb1+/+ and Nfκb1-/- mice without the recall challenge; 2-way ANOVA and unpaired t-test, N=5/group. **(C-E)** Total lung ILCs and ICOS+ST2+ILC2s **(C)** IL5+ and IL13+ ILC2s **(D)**, CD4+, and IL5+ and IL13+ CD4 T cells **(E)**. Unpaired t-test, N=5/group. **(F-H)** Panels F-H compare memory-associated gene expression from lung samples obtained before (**Figure 5A**) and after (**Figure 1A**) the recall challenge and labeled as BR (before recall) and AR (after recall) respectively. The repression program genes– Zeb1, Nr4a2, Bach2, JunD, Fra1 and Fra2 (**Figure 5**, **F-H**) and the preparedness program genes–Fhl2, Mpp7, Stat6, and Srebf2 (**Figure 5**, **I, J**). *P<0.05, **P<0.001 and ***P<0.0001, 2way ANOVA and t test as indicated by the bar above N=6-10/group. These data are representative of 2 independent experiments. ns, not significant.

Next, we examined the importance of NFκB1 in ILC2 memory formation. We intranasally sensitized Nfκb1+/+ (littermate) and Nfκb1-/- female mice with Alt and studied them 3 weeks later without a recall allergen challenge and thereby, avoiding the elicitation of the effector function as shown in **Figure 4A**. Both strains (Nfκb1+/+ and Nfκb1-/-) had the same level of AHR and Siglec F+ CCR3+ eosinophils in the lung (**Figure 4B**; **Figure S5A**). Note that the amplitude of AHR and eosinophilic inflammation was much lower in the absence of the recall allergen challenge. Both strains showed a similar frequency of total ILCs as well as ILC2s cells. However, Nfκb1-/- mice had lower frequency of IL5+ and IL13+ ILC2s (**Figures 4C, D**; **Figure S5B**). The decreased expression of IL5 and IL13 in BAL was confirmed by ELISA (**Figure S5C**). We checked the frequency of CD4 T cells and type-2 cytokine+ CD4 T cells, which were, surprisingly, similar in both strains **(** **Figure 4E**; **Figures S5D, E**).

Next, we examined genes involved in ILC2 memory-associated programs (1)—the gene repression program (Zeb1, Nr4a2, Bach2, JunD, Fra1 and Fra2) (**Figures 4F–H**) and the preparedness program (Fhl2, Mpp7, Stat6, and Srebf2) (**Figures 4I, J**). We studied them at two different time points—after sensitization but before recall in week 6, and after recall in week 7. Most of these genes were elevated before recall during memory formation (**Figures 4F–J**). Nfκb1-/- mice had heightened expression of these genes regardless of the timing of measurement—before or after recall. The results suggested that Nfκb1 was a negative regulator of ILC2 memory-associated gene repression and preparedness programs.

### Effect of NFκB1 null mutation on other members of the NFκB family

**Figure 5:**
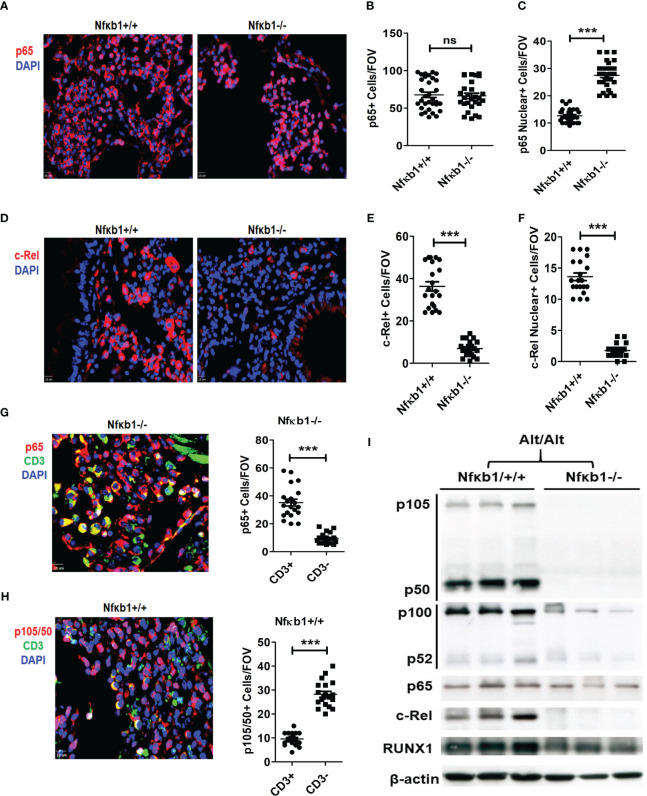
NFκB1 positively regulates all NFkB family members except p65. **(A-C)** Representative immunofluorescence staining of p65 **(A)**, and the quantification of total **(B)** and nuclear **(C)** p65+ cells from Alt/Alt treated Nfκb1+/+ and Nfκb1-/- mice. **(D-F)** Immunofluorescence staining of c-Rel and the quantification of total and nuclear c-Rel+ cells. **(G, H)** Double immunofluorescence staining for CD3 and p65 or p105/50 and the quantification of p65+CD3+, p65+CD3-, p105/50+CD3+ and p105/50+CD3- cells. **(I)** Western blot of the lung tissue from Alt/Alt treated Nfκb1+/+ and Nfκb1-/- mice for NFκB family members. These data are representative of 3 independent experiments with 4-5 mice/group. ***P<0.0001. FOV: Field of View. ns, not significant.

We performed immunofluorescence staining for p65, c-Rel and RelB on the lung tissue from Nfκb1-/- and Nfκb1+/+ mice. Both strains showed a similar frequency of p65+ cells but Nfκb1-/- mice had increased p65 nuclear localization (**Figures 5A–C**). RelB immunostaining was negative in both groups (not shown). c-Rel immunostaining was detected in both groups. However, Nfκb1-/- had less c-Rel+ cells and reduced nuclear localization when compared with Nfκb1+/+ (**Figures 5D–F**). Nfκb1-/- mice showed higher levels of nuclear localization of p65 but surprisingly, no inflammation. To investigate this further, we performed double immunofluorescence staining for CD3 and p65 or p105/50. p65+ cells were mostly CD3+ whereas p105/50+ cells were mostly negative for CD3 (**Figures 5G, H**). Similar to p65, c-Rel+ cells were mostly CD3+ (**Figures S6A, B**). We did western blot of the lung tissue from Alt/Alt mice for p105/50, p100/52, p65, c-Rel and RUNX1. The expression of RelB, c-Rel, and 105/50, was mostly absent but that of p65 was marginally altered in the lung tissue from Nfκb1-/- mice (**Figure 5I**). The expression of RUNX1 and p100/52 was reduced. The foregoing data suggested a differential expression pattern of NFκB family members by ILC2s and CD3 T cells. NFκB1 positively regulated the expression of most of the NFκB family members except that of p65.

### NFκB1 forms a heterodimer with RUNX1 in ILCs in memory-induced asthma

**Figure 6:**
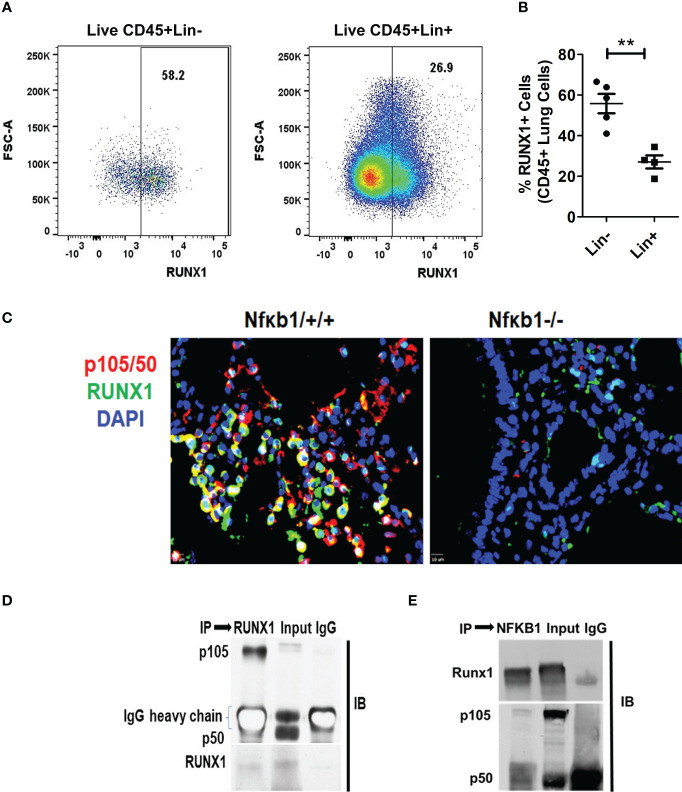
RUNX1 expression in ILCs and heterodimerization with NFκB1. **(A, B)** The frequency of RUNX1+ cells in Lin-(CD45+NK1.1-FcϵR1α- Lin-) cells and Lin+ (CD45+) cell populations. **P<0.001, N=5 per group. **(C)** Co-localization of p105/50 and RUNX1 in the lung tissue from Alt/Alt treated Nfκb1+/+. Nfκb1-/- mice were used as controls. **(D, E)** Co-immunoprecipitation of p105/50 and RUNX1. RUNX1 co-precipitated with p105/50 **(D)** and conversely, p105/50 co-precipitated with RUNX1 **(E)**. IP, immunoprecipitation and IB, immunoblot.

RUNX1 expression by ILC2s was low in Nfκb1-/- mice (**Figure S4C**). We compared the expression of RUNX1 between Lin+ and Lin- cells from Nfκb1 sufficient mice. The frequency of RUNX1+ cells was much higher in the Lin- as compared to the Lin+ cell population (**Figures 6A, B**). We did not observe any difference in RUNX1 expression in Lin+ cells between the Nfκb1+/+ and Nfκb1-/- mouse strains (**Figures S6C, D**). We studied co-localization of p105/50 and RUNX1 by immunofluorescence staining in the lung tissue from Alt/Alt sensitized Nfκb1+/+ and Nfκb1-/- (as a control) mice. RUNX1 and p105/50 were co-localized in Nfκb1+/+ mice, which was expectedly absent in Nfκb1 -/- mice (**Figure 6C**). The heterodimer of RUNX1 and p105/50 functions as a transcriptional activator (17). To examine if heterodimers were present, we performed co-immunoprecipitation experiments with p105/50 and RUNX1. RUNX1 co-precipitated with p105/50 and conversely, p105/50 co-precipitated with RUNX1 (**Figures 6D, E**). We speculate that NFκB1 positively regulated the effector phase of memory ILC2-induced asthma by heterodimerizing with RUNX1.

## Discussion

In this paper, we attempted to dissect the mechanistic processes involved in ILC2 memory formation and memory-driven effector function. We previously reported that ILC2 memory formation was associated with two programs—a gene repression program and a preparedness program. As mentioned previously, Nfkb1 functions as a repressor and a transcriptional activator in its homo- and hetero-dimeric forms, respectively. Hence, it could function in the gene repression program and the preparedness program in the memory model. Using Nfκb1 knockout mice we demonstrated that NFκB1 was essential for the effector phase. Effector and memory processes have an “ying-yang” –interdependent antagonistic relationship. By executing the effector phase, NFκB1 opposed the memory phase gene repression and preparedness programs and downregulated the expression of their genes. NFκB1 executed the effector phase by inducing IL33 and activating ILC2s. NFκB1 was essential for generation of ILC2s, production of type-2 cytokines and induction of allergic inflammation *in vivo*. In this regard the role of NFκB1 in ILC2s was similar to that reported for human NK cells where NFκB1 regulated the effector function (4, 18).

The deletion of Nfκb1 had some additional effects. The Nfκb1germline deletion resulted in impaired expression of the NFκB family members except p65. Nfκb1-/- mice had reduced RUNX1 expression. Interestingly, NFκB1 and RUNX1 were expressed at a very high level in ILC2s as compared to Lin+ non-ILC2 immune cells. They also formed a heterodimer. Preferential expression of NFκB1 and RUNX1 in ILC2s implied a non-canonical heterodimer-mediated execution of the ILC2 effector function in our asthma model.

Previously NFκB1 deficiency was shown to inhibit Th2 cells and allergic inflammation in a Th2-dependent model of asthma, which involved percutaneous sensitization with an adjuvant (12). We did not observe a significant Th2 cell involvement in our model likely to due to the difference in the method of sensitization and the recall challenge. There was no difference in IL5+ and IL13+ CD4 T cells between Nfκb1+/+ and Nfκb1-/- mice. However, the total number of CD4 T cells recovered from the lung was reduced in Nfκb1-/- mice. The latter mice had reduced expression of ICAM1. We believe that the reduced recovery of CD4 T cells was due to reduced vascular permeability and inflammatory cell influx. Nfκb1-/- mice had reduced expression of Treg (Foxp3+CD25+CD4) cells in our model. This is in line with a study that demonstrated a non-redundant role of NFκB1 in development and maintenance of effector T regulatory (eTreg) cells in mice and humans (19).

The transcription factor GATA3 is known to regulate the development and function of Th2 cells and ILC2s (20–22). Nfκb1 was previously shown to regulate GATA3 expression in Th2 cells in allergic airway inflammation (12). In our study GATA3 expression in ILC2s and CD4 T cells was similar in Nfκb1+/+ and Nfκb1-/- mice (**Figure 3D**; **Figure S4B**). In agreement with our finding, Serre et al. reported that OVA-specific CD4 T cells had unchanged GATA3 expression in Nfκb1-/- as compared to Nfκb1+/+ mice (23). The foregoing results suggested that the transcription of type-2 cytokine genes was regulated by additional transcription factors. Like Nfκb1 deficient ILC2s, amphiregulin (AREG) deficient ILC2s had impaired type-2 cytokine production despite having a wild-type level of GATA3 expression (24). RUNX1 was recently reported to regulate type-2 cytokine production in ILC2s (16). We found that Nfκb1-/- mice had reduced expression of RUNX1 (**Figure S4C**). RUNX1 participates in regulation of the NFκB signaling pathway through interaction with either the IκB kinase complex in the cytoplasm or the NFκB1 subunit p50 in the nucleus and is important for the inflammatory response in the lung (25, 26). We found RUNX1-p105/50 heterodimers and their co-localization in the nucleus in our asthma model. We speculate that the RUNX1-p105/50 heterodimer functioned as a transcriptional activator of type-2 cytokine genes in ILC2s, and that of IL33 in epithelial cells in the lung. Note that we were unable to perform co-immunoprecipitation studies using isolated ILC2s due to the low cell number. For this reason we could not conclude in a definitive manner that RUNX1-p150/50 heterodimers occurred in ILC2s.

Reduced expression of IL33 in Nfκb1-/- mice in our model is in agreement with a recent report on lower levels of IL33 expression in Nfκb1-/- mice after allergen challenge. The latter was associated with reduced airway inflammation (27). TSLP and IL-33 reciprocally promote each other’s expression to enhance innate type-2 airway inflammation (28). Accordingly, we observed reduced TSLPR+ ILC2 in Nfκb1-/- mice. ICAM1 regulates inflammatory cell influx into the airways from the blood. The expression of ICAM1 is elevated in asthma (29–31). ICAM1 expression was reduced in Nfκb1-/- mice (**Figure S4H**). Of interest, Yang et al. did not find any difference in ICAM1 expression in Nfκb1-/- mice (32) and the reason for this discrepancy is unclear.

In Nfκb1-/- mice, Nfκb2 and c-Rel were reduced and p65 was unchanged. Interestingly, there was a preferential nuclear localization of p65 in CD3+ cells from Nfκb1-/- as compared to Nfκb1+/+ mice, which is in agreement with another report (33). Reduced expression of c-Rel might have contributed to reduced Treg cells in Nfκb1-/- mice (34). Nfκb2 is important for activation of T and NK cells (35). Cells from Nfκb2-/- mice that lacked p52, formed RelB:p50 dimers instead, and compensated for the loss of RelB:p52 activity. Similarly, cells that lacked Nκkb1, formed p65:p52 NFκB dimers instead, with almost the same level of p65 activation and target inflammatory gene expression (36). The present study suggested that Nfkb1 acted as an upstream regulator of Nfκb2 and c-Rel. Consequently, despite the presence of sufficient p65, Nfκb1-/- mice did not show airway inflammation. c-Rel and p65+ cells were mostly CD3+ whereas as Nfκb1+ cells were mostly CD3- small lymphoid cells in our model suggesting a dominant role of NFκB1 in ILC2s.

In conclusion, NFkB1 was essential for the effector phase of ILC2 memory and induction of memory-driven asthma in the mouse model. On the other hand, NFκB1 opposed memory formation and downregulated the memory-associated gene repression and preparedness programs. The latter pointed to an interdependent antagonism between the memory and the effector processes of ILC2 function. We believe that a dynamic balance between these two processes is essential for an optimal memory-driven response in host defense.

## Data availability statement

The original contributions presented in the study are included in the article/**Supplementary Material**. Further inquiries can be directed to the corresponding author.

## Ethics statement

The animal study was reviewed and approved by National Jewish Health IACUC.

## Author contributions

MV conducted the all of the experiments, contributed to the design of experiments, analyzed and prepared the data, and wrote the original draft of manuscript. DV helped him in experiments, data analysis, and manuscript preparation. ASS, KS, RV and AS helped MV in some mouse experiments. RA conceived the concept, designed the experiments, analyzed the data, and reviewed and edited the manuscript. All authors contributed to the article and approved the submitted version.

## Supplementary material

Supplementary Figure 1
**(A–C)** The number of ILCs and flow cytometry gating strategy for isolation of lung ILCs. **(A)** Absolute number of total ILCs, ILC2s, IL5+ and IL13+ ILCs from Alt/Alt and Sal/Alt treated WT B6 mice. ***P<0.0001 and **P<0.001, N=4/group. **(B)** The gating strategy for ILCs: ILCs (CD45+Lin-CD25+NK1.1- FcϵR1α-), ILC2s (CD45+Lin-CD25+NK1.1- FcϵR1α-ICOS+ ST2+), IL5+ and IL13+ ILC2s. **(C)** FMO (florescence minus one) for ST2, ICOS, Il5 and IL13. **(D–F)** Gating strategy for eosinophils **(D)**, NFκB1 expressing ILC2s **(E)** and Lin- and Lin+ cells **(F)**.

Supplementary Figure 2 The expression of NFκB1 (p105/50) among different lymphoid cell populations. **(A, B)** Representative flow plots showing the frequency of p105/50+ cells in NK (NK1.1+), ILC1(T-bet+), ILC3 (RoRγt+) and ILC2 (GATA3+) cells; All ILCs were gated as CD45+ Lin-NK1.1-FcϵR1α-CD25+. **(C)** Representative flow plot for the expression of p105/50 in epithelial(CD45-Ep-Cam+) and CD4+(CD45+) cells. **(D)** The frequency of p105/50+ cells in all studied cell types. ***P<0.0001, N=4/group.

Supplementary Figure 3 The flow cytometry gating strategy and experimental data from the male mice. **(A)** The gating strategy and representative flow plots for eosinophils and neutrophils from the lung digest obtained from Alt/Alt treated Nfκb1+/+ and Nfκb1-/- mice. **(B)** The gating strategy for ILCs (CD45+Lin-CD25+NK1.1- FcϵR1α-), ILC2s (CD45+Lin-CD25+NK1.1- FcϵR1α-ICOS+ ST2 and IL5/13+ ILC2s. **(C–G)** Data from experiments done with the male mice. Airway hyperactivity **(C)**, lung eosinophils **(D)**, lung inflammation, PAS staining for mucus, and Trichrome staining for collagen deposition **(E)**, total ILCs, ILC2s, IL5+ and IL13+ ILC2s **(F)**, and IL5 and IL13 levels in BAL **(G)**. *P<0.05, **P<0.001 and ***P<0.0001, 2way ANOVA and t test, N=4-5/group. These data are representative of 2 independent experiments.

Supplementary Figure 4 Representative ILC data from the naïve mice, and NFκB1 regulation of type-2 inflammation-associated molecules. **(A)** Representative flow plots showing the frequency of CD4+ T cells and CD25+, ST2+, ICOS+ and GATA3+ ILC2s in naïve Nfκb1+/+ and Nfκb1-/- mice. Eosinophils and neutrophils from Nfκb1+/+ and Nfκb1-/- Alt/Alt mice. **(B)** GATA3 expression and MFI in ILC2s and CD4 T cells from Nfκb1+/+ and Nfκb1-/- Alt/Alt mice, N=4-5/group. **(C)** RUNX1+ILC2s (CD45+NK1.1-FcϵR1α- Lin-CD25+ ICOS+ST2+) in Alt/Alt mice. p. ***P<0.0001, N=4-5/group. **(D)** qPCR analysis of mRNA for IL25 and TSLP from the lung tissue from Alt/Alt treated Nfκb1+/+ and Nfκb1-/- mice; N=8-10/group. **(E)** Expression of TSLPR+ILC2s (CD45+NK1.1-FcϵR1α- Lin-CD25+ ICOS+ST2+). **P<0.001, N=4/5group. (**F, G**) IL33 mRNA expression **(F)** and representative immunofluorescence staining and quantification of IL33 protein expression **(G)** in the lung from Alt/Alt-treated mice. **P<0.001 and ***P<0.0001 (FOV: Field of View), N=8/group. **(H)** Representative immunofluorescence staining of ICAM1 in the lung and quantification. ***P<0.0001, N=4-5/group. All data are representative of 3 independent experiments.

Supplementary Figure 5 Representative data from Alt-treated mice obtained before the recall challenge. **(A)** Representative flow plots showing eosinophils in the lung from Alt -treated mice examined before the recall challenge. **(B)** Representative flow plots for IL5/IL13+ cellsILC2s. **(C)** BAL IL5 and IL13 levels (ELISA) done before the recall challenge. **(D, E)** Representative flow plots for CD4+ T cells, and IL5/IL13+ CD4+ T cells in the lung examined before the recall challenge.

Supplementary Figure 6
**(A, B)** Representative images from double immunofluorescence staining for CD3 and c-Rel, and the quantification of c-Rel+ cells in CD3+ and CD3- cell populations. Representative flow plots of RUNX1 expression in lung Lin+ cells from Nfκb1+/+ and Nfκb1-/- mice **(C)** and their quantification **(D)**.

Supplementary Table 1 Name of primers.
