# Supplementary figures and images for: NFκB1 inhibits memory formation and supports effector function of ILC2s in memory-driven asthma
Source: Front Immunol. 2023 Jul 27;14:1217776. doi: 10.3389/fimmu.2023.1217776 (PMC10415221; doi:10.3389/fimmu.2023.1217776)

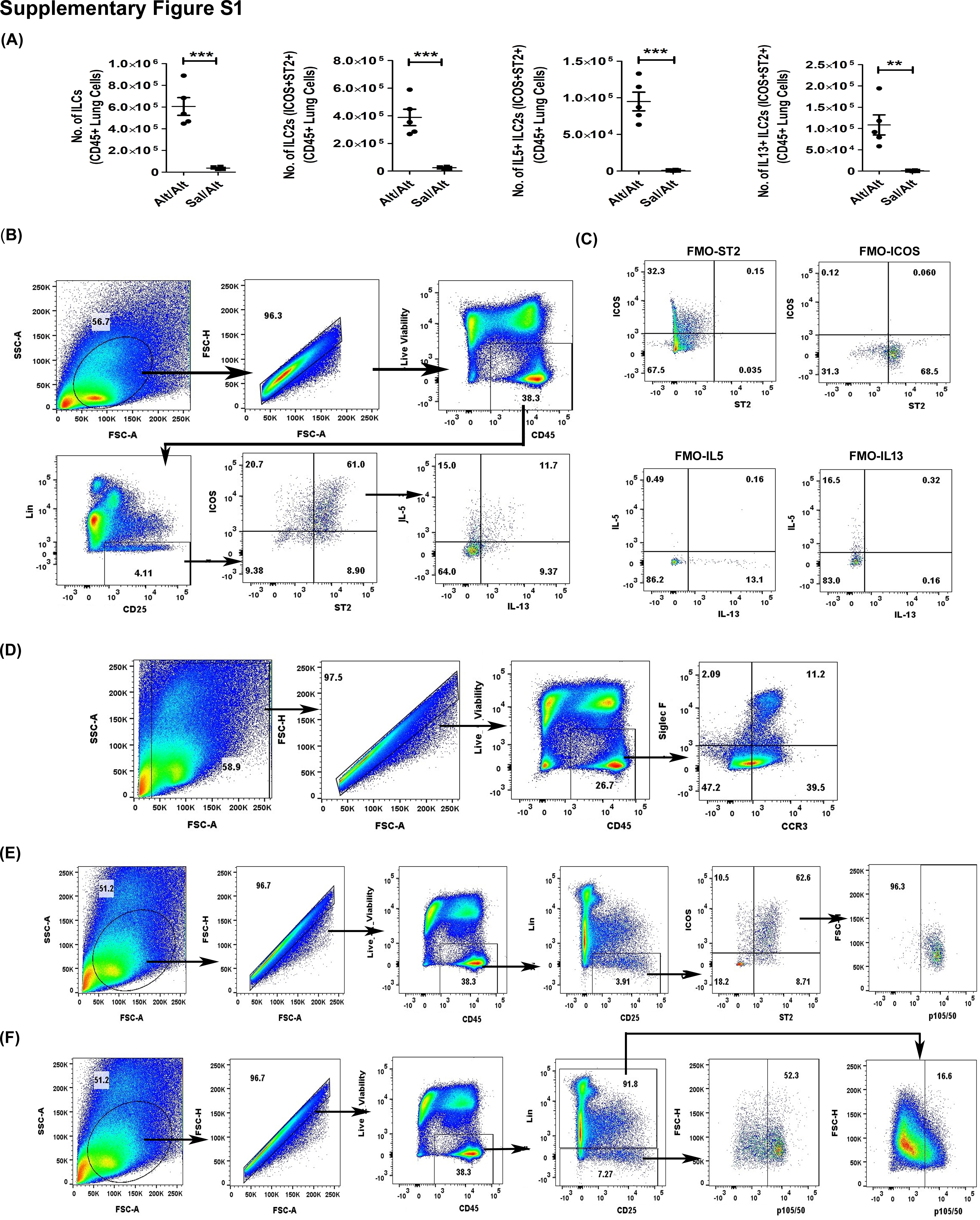

Supplement: Supplementary Figure 1 — (A–C) The number of ILCs and flow cytometry gating strategy for isolation of lung ILCs. (A) Absolute number of total ILCs, ILC2s, IL5+ and IL13+ ILCs from Alt/Alt and Sal/Alt treated WT B6 mice. ***P<0.0001 and **P<0.001, N=4/group. (B) The gating strategy for ILCs: ILCs (CD45+Lin-CD25+NK1.1- FcϵR1α-), ILC2s (CD45+Lin-CD25+NK1.1- FcϵR1α-ICOS+ ST2+), IL5+ and IL13+ ILC2s. (C) FMO (florescence minus one) for ST2, ICOS, Il5 and IL13. (D–F) Gating strategy for eosinophils (D), NFκB1 expressing ILC2s (E) and Lin- and Lin+ cells (F). [file Image_1.jpeg]

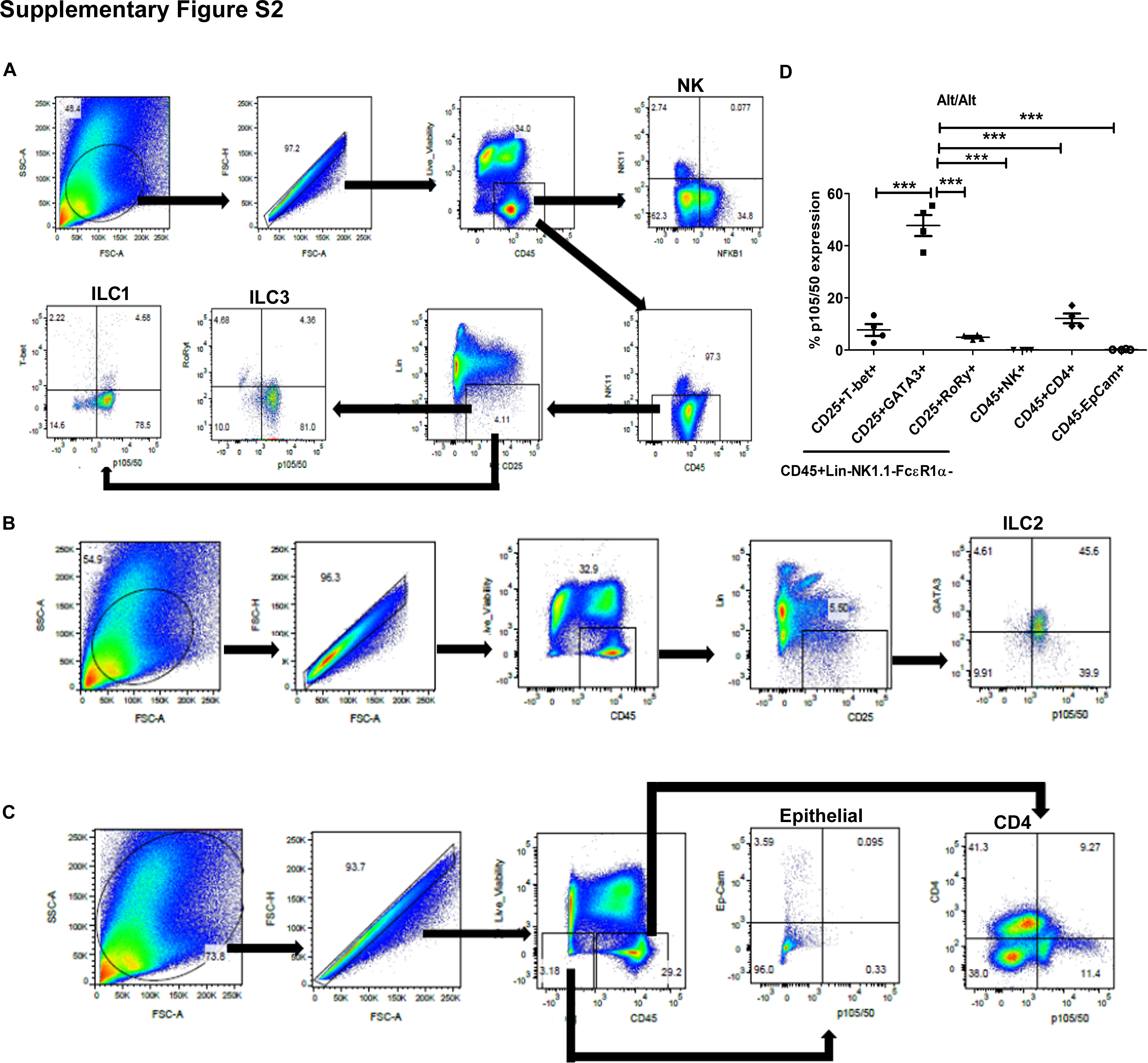

Supplement: Supplementary Figure 2 — The expression of NFκB1 (p105/50) among different lymphoid cell populations. (A, B) Representative flow plots showing the frequency of p105/50+ cells in NK (NK1.1+), ILC1(T-bet+), ILC3 (RoRγt+) and ILC2 (GATA3+) cells; All ILCs were gated as CD45+ Lin-NK1.1-FcϵR1α-CD25+. (C) Representative flow plot for the expression of p105/50 in epithelial(CD45-Ep-Cam+) and CD4+(CD45+) cells. (D) The frequency of p105/50+ cells in all studied cell types. ***P<0.0001, N=4/group. [file Image_2.jpeg]

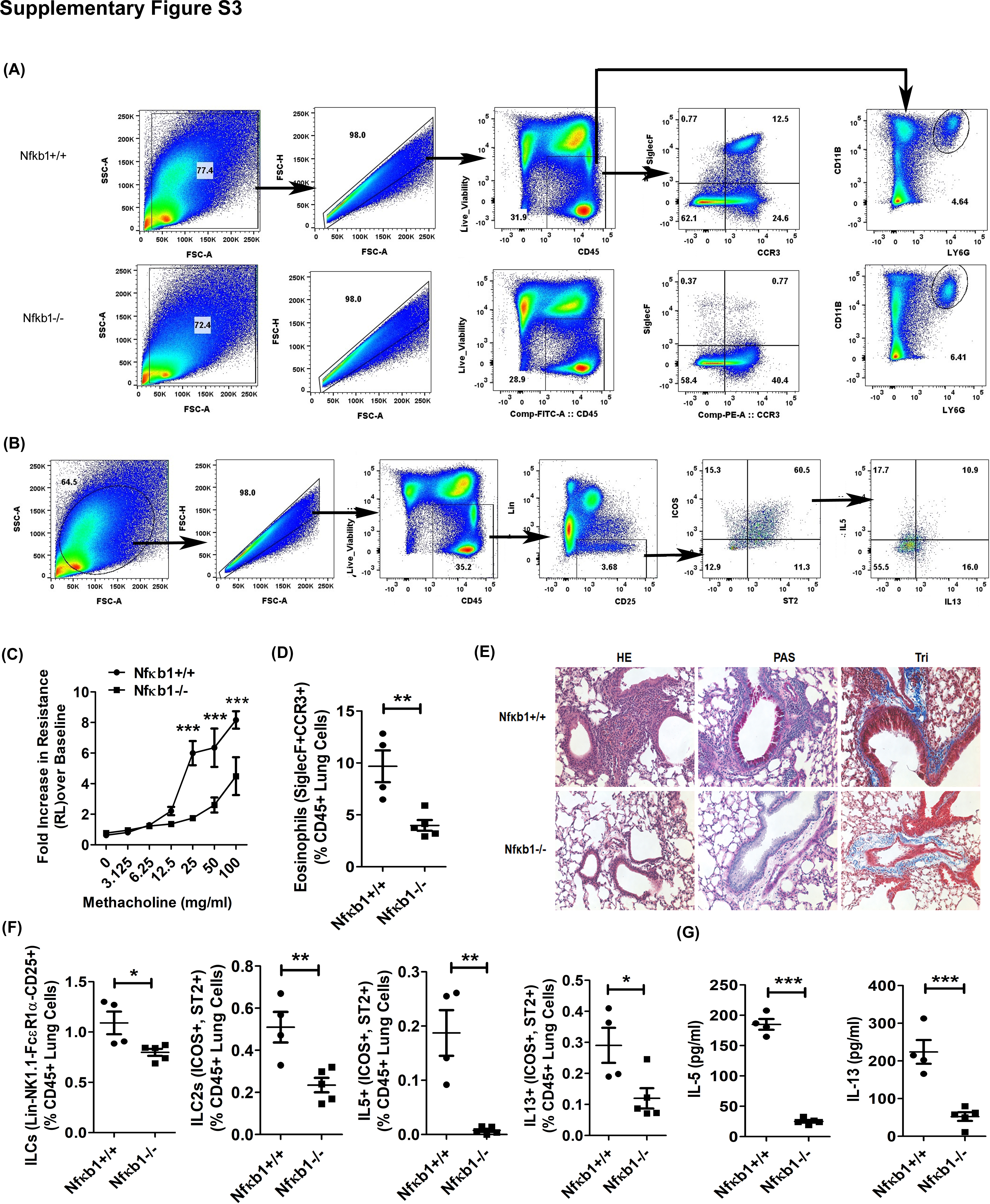

Supplement: Supplementary Figure 3 — The flow cytometry gating strategy and experimental data from the male mice. (A) The gating strategy and representative flow plots for eosinophils and neutrophils from the lung digest obtained from Alt/Alt treated Nfκb1+/+ and Nfκb1-/- mice. (B) The gating strategy for ILCs (CD45+Lin-CD25+NK1.1- FcϵR1α-), ILC2s (CD45+Lin-CD25+NK1.1- FcϵR1α-ICOS+ ST2 and IL5/13+ ILC2s. (C–G) Data from experiments done with the male mice. Airway hyperactivity (C), lung eosinophils (D), lung inflammation, PAS staining for mucus, and Trichrome staining for collagen deposition (E), total ILCs, ILC2s, IL5+ and IL13+ ILC2s (F), and IL5 and IL13 levels in BAL (G). *P<0.05, **P<0.001 and ***P<0.0001, 2way ANOVA and t test, N=4-5/group. These data are representative of 2 independent experiments. [file Image_3.jpeg]

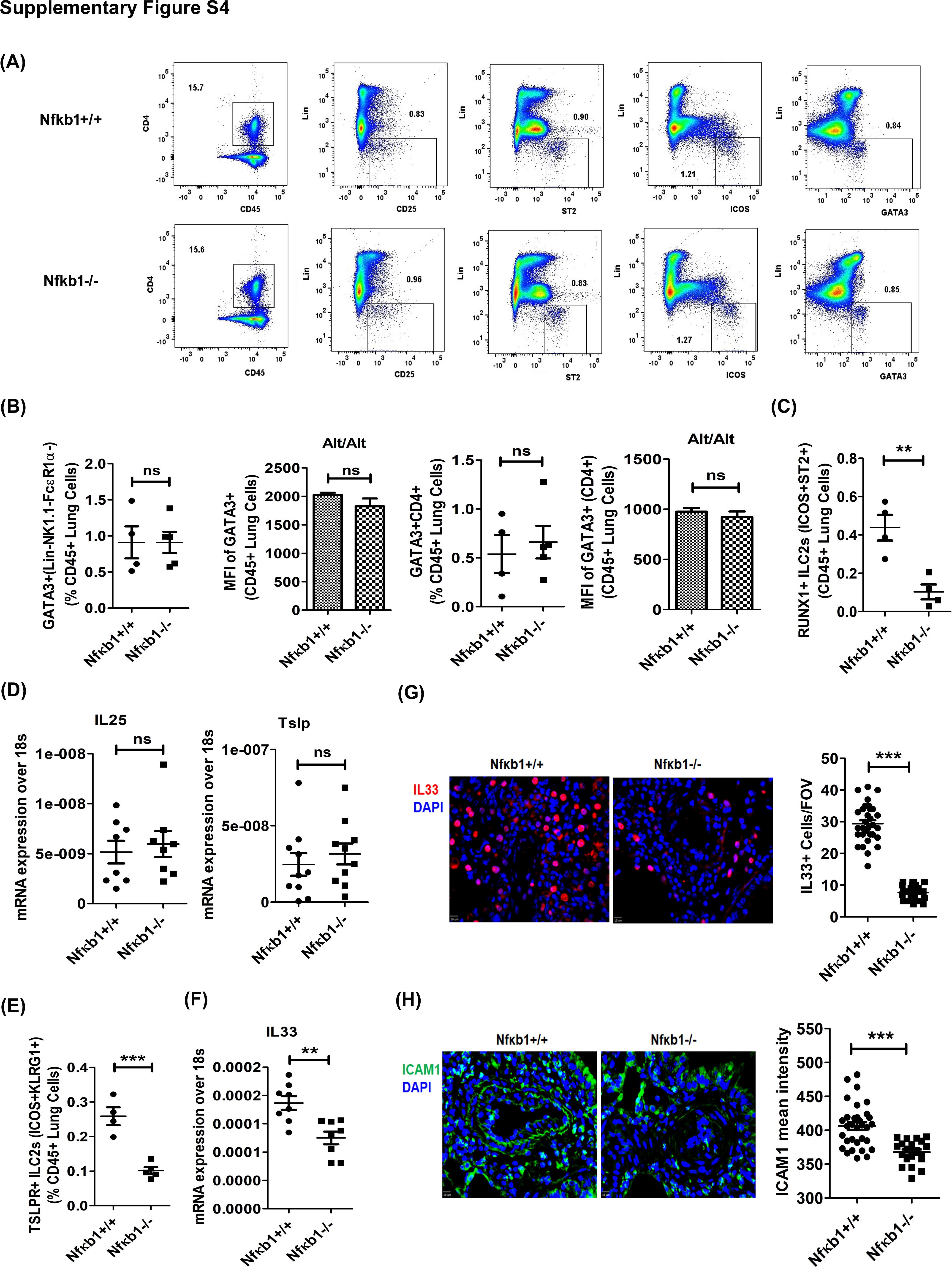

Supplement: Supplementary Figure 4 — Representative ILC data from the naïve mice, and NFκB1 regulation of type-2 inflammation-associated molecules. (A) Representative flow plots showing the frequency of CD4+ T cells and CD25+, ST2+, ICOS+ and GATA3+ ILC2s in naïve Nfκb1+/+ and Nfκb1-/- mice. Eosinophils and neutrophils from Nfκb1+/+ and Nfκb1-/- Alt/Alt mice. (B) GATA3 expression and MFI in ILC2s and CD4 T cells from Nfκb1+/+ and Nfκb1-/- Alt/Alt mice, N=4-5/group. (C) RUNX1+ILC2s (CD45+NK1.1-FcϵR1α- Lin-CD25+ ICOS+ST2+) in Alt/Alt mice. p. ***P<0.0001, N=4-5/group. (D) qPCR analysis of mRNA for IL25 and TSLP from the lung tissue from Alt/Alt treated Nfκb1+/+ and Nfκb1-/- mice; N=8-10/group. (E) Expression of TSLPR+ILC2s (CD45+NK1.1-FcϵR1α- Lin-CD25+ ICOS+ST2+). **P<0.001, N=4/5group. (F, G) IL33 mRNA expression (F) and representative immunofluorescence staining and quantification of IL33 protein expression (G) in the lung from Alt/Alt-treated mice. **P<0.001 and ***P<0.0001 (FOV: Field of View), N=8/group. (H) Representative immunofluorescence staining of ICAM1 in the lung and quantification. ***P<0.0001, N=4-5/group. All data are representative of 3 independent experiments. [file Image_4.jpeg]

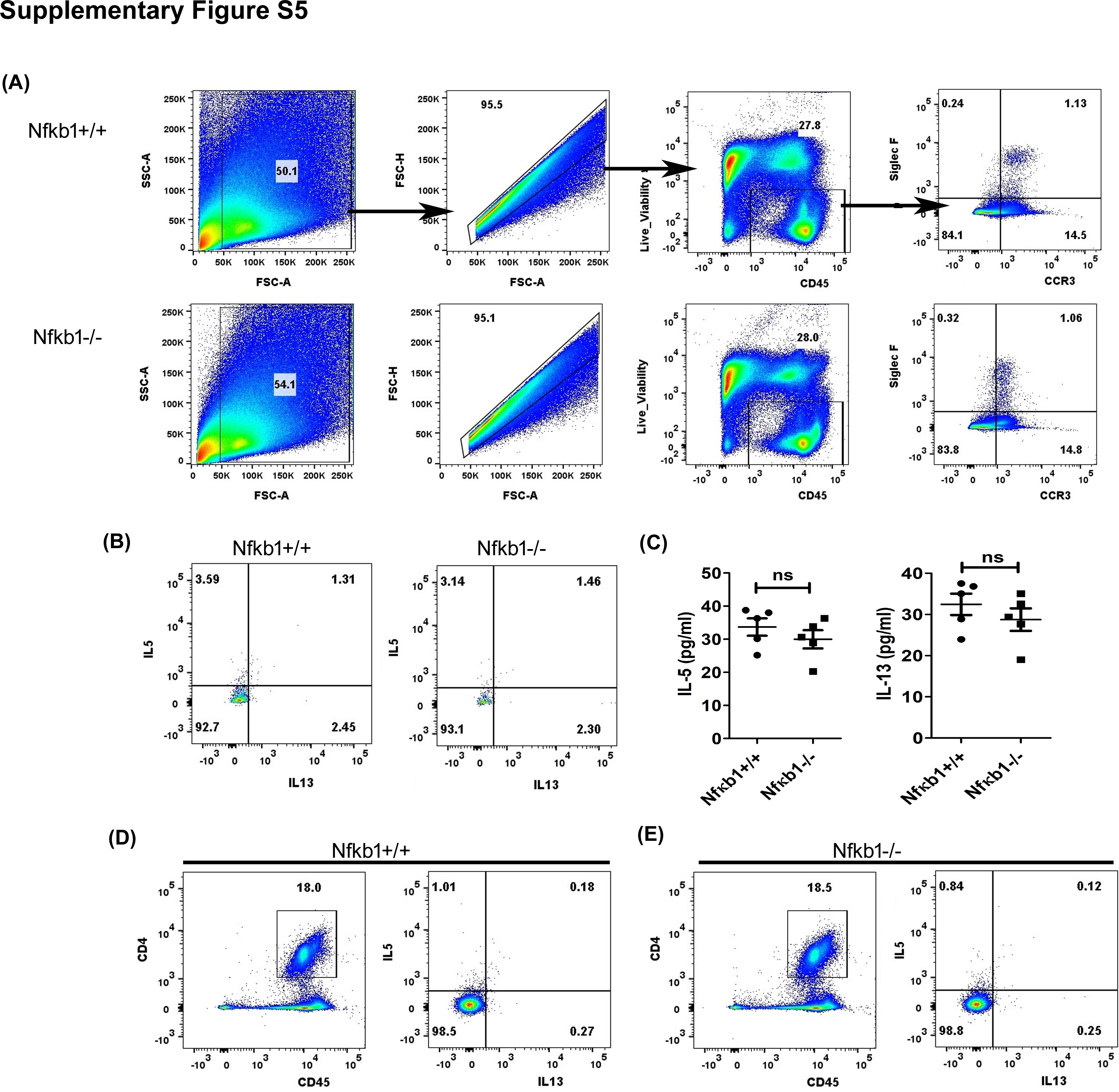

Supplement: Supplementary Figure 5 — Representative data from Alt-treated mice obtained before the recall challenge. (A) Representative flow plots showing eosinophils in the lung from Alt -treated mice examined before the recall challenge. (B) Representative flow plots for IL5/IL13+ cellsILC2s. (C) BAL IL5 and IL13 levels (ELISA) done before the recall challenge. (D, E) Representative flow plots for CD4+ T cells, and IL5/IL13+ CD4+ T cells in the lung examined before the recall challenge. [file Image_5.jpeg]

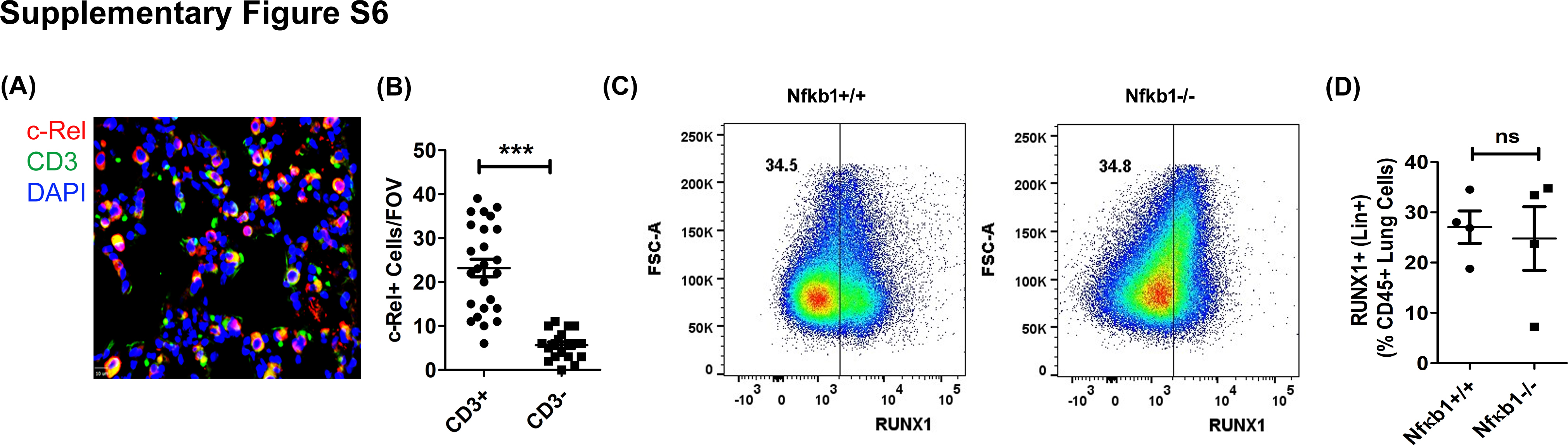

Supplement: Supplementary Figure 6 — (A, B) Representative images from double immunofluorescence staining for CD3 and c-Rel, and the quantification of c-Rel+ cells in CD3+ and CD3- cell populations. Representative flow plots of RUNX1 expression in lung Lin+ cells from Nfκb1+/+ and Nfκb1-/- mice (C) and their quantification (D). [file Image_6.jpeg]
